# The Relationship between Physical Activity, Physical Fitness, Cognition, and Academic Outcomes in School-Aged Latino Children: A Scoping Review

**DOI:** 10.3390/children11030363

**Published:** 2024-03-19

**Authors:** J. P. Marrero-Rivera, Olivia Sobkowiak, Aimee Sgourakis Jenkins, Stefano J. Bagnato, Christopher E. Kline, Benjamin DH Gordon, Sharon E. Taverno Ross

**Affiliations:** 1 Department of Health and Human Development, School of Education, University of Pittsburgh, Pittsburgh, PA 15260, USA; jpm190@pitt.edu (J.P.M.-R.);; 2 Department of Research, Learning and Media, University Library System, University of Pittsburgh, Pittsburgh, PA 15260, USA

**Keywords:** pediatrics, physical activity, child development, academic achievement, cognition

## Abstract

This scoping review provides an overview of the relationship between physical activity, physical fitness, cognition, and academic outcomes in Latino school-aged children and identifies areas for future research. A primary search was conducted in PubMed, PsycINFO, Web of Science, and ERIC for original-research articles meeting the inclusion criteria; the search results were uploaded into PICO Portal and assessed by two independent reviewers. Of the 488 initial search results, 50 articles were eligible for full-text review, and 38 were included in this review. Most studies were cross-sectional, conducted in the United States or Chile, and included children 5–18 years old. Overall, the majority of articles reported positive associations between physical activity or physical fitness and cognitive outcomes (*n* = 11/12; 91.7%), and physical activity or physical fitness and academic outcomes (*n* = 22/28; 78.6%). In sum, this review provided consistent evidence for higher amounts of physical activity and greater physical fitness to be associated with various positive cognitive and academic outcomes in a school-aged Latino population. This scoping review also elucidated a substantial gap in the research regarding study design, with a discernible lack of interventional efforts. Future studies should test physical activity interventional strategies to optimize cognitive and academic outcomes in school-aged Latino populations.

## 1. Introduction

Participation in physical activity is vital for school-aged children, given its reported benefits for cognitive function and cardiovascular health [1]. Additionally, physical activity is reported to elicit benefits for bone health, weight status, and muscular fitness—all of which are crucial dimensions of child development [2]. The World Health Organization [3] recommends that school-aged children, ages 5 through 17, participate in at least 60 min of moderate-to-vigorous physical activity daily for optimal health. Despite this recommendation, up to 81% of school-aged children—globally—are reported to be insufficiently physically active [4]. Physical inactivity in school-aged children is a multi-faceted issue, with many barriers contributing to the lack of physical activity seen across the globe. 

For many school-aged children of marginalized and varying cultural backgrounds, the social determinants of health play a key role in the level of engagement in physical activity. Previous studies have linked unhealthy housing, unsafe neighborhoods, and fewer places to participate in safe physical activity with lower overall physical activity levels, though living in metropolitan areas appears to increase the odds of young Latino children engaging in moderate-to-vigorous physical activity in Latin American countries, when living in built environments that have spaces within 20 min of walking and/or 30 min using public transportation [5,6]. This is a concerning matter for Latino children across the globe, as a lack of sufficient physical activity during this period of life has implications for poorer health outcomes, substantial decrements to cognitive development, and poorer academic outcomes [7,8,9]. 

Ultimately, inactive children tend to become inactive adults [10]. Given that school-aged Latino children across the globe are engaging in less activity resulting from the aforementioned barriers, special attention to this population is merited. This is especially the case for school-aged Latino children living in Latin American countries, as Latin American adults are reported to have the highest prevalence of inactivity compared to adults of other countries [4]. This serves as a driving force behind this work, given that prior reviews have not thoroughly evaluated these variables in school-aged Latino children from a global perspective. Future efforts should focus on increasing physical activity to aide in ameliorating the present disparities that school-aged Latino children face across the globe. This work can provide information to enhance policies which reap physical health and cognitive benefits for this population—which may yield additional benefit(s) in academic outcomes.

Cognition, defined as a range of information-processing skills involved in learning, memory, communication, and problem solving, can be divided into six main constructs [11]. These include executive function, episodic memory, language skills, processing speed, spatial and numerical processing, and social cognition. Executive functioning, episodic memory, and language skills are cognitive constructs that are particularly pertinent to school-aged children’s overall academic and developmental outcomes. It is understood that improvements in cognition stemming from physical activity can be explained through favorable changes in cerebral blood flow and the upregulation of circulating growth factors, neurotransmitters, and neurotrophins. Ultimately, these changes lead to enhanced synaptic plasticity and neurogenesis [12,13]. In essence, the structures of a child’s developing brain are exposed to a better production and delivery of crucial chemicals and hormones that optimize the growth and function of neural tissue when a child is physically active. While these augmentative effects of physical activity on cognition are certainly well-researched for some of the cognitive constructs, others remain understudied in their relation to physical activity in a Latino school-aged population. 

Much of the knowledge base regarding physical activity and cognition in children centers on executive function and its relationship with physical activity. Data from cross-sectional studies and randomized controlled trials have indicated that physically active children possess greater executive function than their inactive counterparts [14,15], and interventional studies have indicated favorable changes in attributes of executive function (e.g., creativity) in response to varying forms of physical activity via physical education [16]. However, very few studies on physical activity and executive function have focused on a Latino school-aged population. This population should be at the forefront of this work, as many Latino children are bilingual and language skills such as bilingualism are reported to have protective influences on the development and capabilities of executive function [17]. 

Interestingly, the effects of bilingualism can last throughout the lifespan and the benefits of bilingualism may even extend into episodic memory [18]. While episodic memory is reported to improve with increasing physical activity in older adults, there is conflicting evidence for the relationship between physical activity and episodic memory in younger populations [19]. This is likely explained by episodic memory undergoing intensive development beginning at the age of seven, with much of the development and capabilities of episodic memory specific to the child and their lived experiences [20]. No two children will have similar developmental changes and function of episodic memory, which may make the assessment of episodic memory in school-aged children relatively challenging and not easily elucidated. Despite this, further efforts are required to understand whether there are associations between physical activity and episodic memory in Latino school-aged children.

Regardless of the cognitive construct in question, improvements in cognition stemming from physical activity engagement may transcend into other domains of interest for school-aged children—including academic outcomes (e.g., academic performance, academic achievement). Evidence from randomized controlled trials suggests that physical activity mediates improvements to various cognitive constructs, which then may contribute to improved academic outcomes—especially when the physical activity is accompanied with cognitively engaged exercises [21]. This is supported by evidence from systematic reviews and meta-analyses agreeing that physical activity can potentially have positive effects on academic performance via greater cognitive capabilities [22,23]. However, some of the evidence suggests that the correlations between physical activity and academic outcomes are weak [24,25]. 

Higher intensity levels and greater durations of physical activity are reported to elicit better academic outcomes [26]. Additionally, there are other variables in a child’s life that contribute to academic outcomes (e.g., friendships in both school and in extracurricular activities). These variables may improve a child’s sense of belonging in a school setting, which can also improve academic outcomes [27]. Nevertheless, many of these reviews did not focus on a Latino school-aged population, which is a concerning matter given that Latino school-aged children are at a higher risk of decreased academic achievement compared to non-Latino children within the United States [28]. Similarly, school-aged Latino children in Latin American countries face extensive educational gaps, with recent indications of general downward trends and an overall lower position in academic performance and international ranking [29].

In sum, school-aged Latino children across the globe are less physically active than their racial/ethnic counterparts. Several factors contribute to this, but many are rooted in barriers linked to the social determinants of health. These barriers to physical activity have implications for decreased physical health, cognition, and academic performance. Though many Latino school-aged children may have advantages in cognitive function due to their bilingualism, the level of protection from the physical and mental effects related to physical inactivity is unclear. This scoping review will provide an overview of the relationship between physical activity, physical fitness, cognition, and academic outcomes in a Latino school-aged population and identify areas for future research.

### Objectives

We conducted a scoping review to report on the associations between physical activity or physical fitness and cognitive or academic outcomes in school-aged Latino children. This scoping review addresses the following research questions:

1. What is the relationship between physical activity or physical fitness and cognitive outcomes in Latino children aged 6–17?
2. What is the relationship between physical activity or physical fitness and academic outcomes in Latino children aged 6–17?

Research Question #1 (RQ1) addressed both the physiological and psychological influences that physical activity or physical fitness has on overall cognitive function, with a specific interest in executive function, episodic memory, and language skills. Research Question #2 (RQ2) addressed the overarching effects that physical activity or physical fitness might have on academic outcomes for school-aged Latino children. This included both academic performance and academic achievement.

## 2. Materials and Methods

### 2.1. Protocol and Registration

This review followed the scoping review framework outlined by Arksey and O’Malley (2005) [30] and in accordance with the PRISMA Extension for Scoping Reviews (PRISMA-ScR). A protocol of this scoping review is registered on *Open Science Framework* (https://osf.io/a5m6d accessed on 17 March 2024). 

### 2.2. Eligibility Criteria and Information Sources

Articles were eligible for inclusion if they had samples that included Latino children between 6 to 17 years of age (i.e., school-aged). Articles that were a majority school-aged Latino sample (51%+) met the threshold for inclusion. Articles that had a less than 51% school-aged Latino sample were also included if the number and/or percentage of the population of interest was reported, and if the results specific to the population of interest were also reported. Articles that did not have participants meeting the criteria were excluded from the review.

Articles with physical activity interventions and/or physical activity or physical fitness as the independent variables were included in this review. In the case of articles that did not have a physical activity intervention, they were included under the stipulation that they reported a physical activity-, fitness-, or exercise-related independent variable. Examples of such articles included device-based or self-reported physical activity data, or graded exercise testing results. Articles that did not have either a physical activity intervention or a physical activity-, fitness-, or exercise-related independent variable, such as those mentioned above, were excluded from the review. 

The main outcomes of interest were three cognitive constructs (i.e., executive function, episodic memory, language skills) and academic outcomes (performance and/or achievement). Additional cognitive constructs identified in the search process were also included in this scoping review. To meet the inclusion criteria, these cognitive constructs must have been assessed via cognitive battery tests specific to those in middle childhood up to adolescence. Academic outcomes were reported via standardized examination, academic grades, or other school-based achievement or performance indicators. 

The main domain of knowledge for this review is exercise psychology, where the effects of physical activity are assessed in terms of cognition and academic performance. This encompasses multiple fields of study and includes articles that stemmed from works in public health, biomedical sciences, exercise science, developmental psychology, and education. Given the emphasis on the Latino population, articles were included from all countries under the guideline that there was an English and/or Spanish version of the study available for review. 

The inclusion criteria of this scoping review allowed for various study types. The inclusion criteria were open to—but not limited to—randomized controlled trials, non-randomized experimental studies with or without a control group, cross-sectional studies, prospective cohort (i.e., longitudinal) studies, and case–control studies. Each of these study types was considered when in agreement with the inclusion criteria. Review papers were used only to identify additional original research articles. Protocol papers and methodological papers were excluded, given their lack of reported results. Articles were not limited based on date of publication. Table 1 provides additional details on the eligibility criteria.

### 2.3. Search Strategy

The search strategy was drafted by a university librarian (A.S.J.) and finalized after discussion with the three team members responsible for the initial and full-text review phases (J.P.M-R.; O.S.; S.E.T.R.). Initial searches were conducted in Google Scholar and PubMed between February 2022 and August 2022 to identify keywords and develop a search string. A primary search for relevant articles was conducted in August 2022, and a secondary search for newer and relevant articles was later conducted in April 2023. We searched four online databases for relevant results: PubMed, ProQuest PsycINFO, Web of Science, and ERIC via EBSCOhost. The initial review phase was conducted by two independent reviewers (J.P.M-R. and O.S.) using *PICO Portal*, a systematic review platform that leverages artificial intelligence to accelerate research and innovation [31]. This web-based tool provides an interface for uploading search results, which then utilizes artificial intelligence to remove article duplications across databases, while simultaneously identifying keywords from selected and relevant articles to continue expediting the article selection process. The initial search results were screened utilizing the following domains: population; intervention; comparison; and outcome(s). Conflicts in article selection via eligibility criteria were rectified with a designated adjudicator (S.E.T.R.). Following this, a full-text review phase was conducted to determine the final sample of articles included for this review. Further conflicts in final article selection were rectified with the designated adjudicator. A sample search strategy is available in Table 2. 

### 2.4. Data Items, Synthesis, and Charting 

After ascertaining the final sample of articles, data from the eligible articles were extracted and uploaded into a Microsoft Excel file under the following data categories: author(s); publication year; article title; study type (e.g., cross-sectional, cohort); aims and purpose; population demographics/characteristics; measure of physical activity (e.g., accelerometer data, field testing); cognitive/academic measure(s) (e.g., neuro-cognitive tests, standardized testing, grade point average); and study results. In this process, the articles were grouped by outcome measures (cognitive and/or academic outcomes), in accordance with our two research questions (see below). For this scoping review, results for each research question were presented as articles with evidence for a positive association (e.g., physical activity was associated with better cognition), articles with evidence for a negative association (e.g., physical activity was associated with worse cognition), and articles with no evidence for an association. 

## 3. Results

### 3.1. Search Results

**Figure 1:**
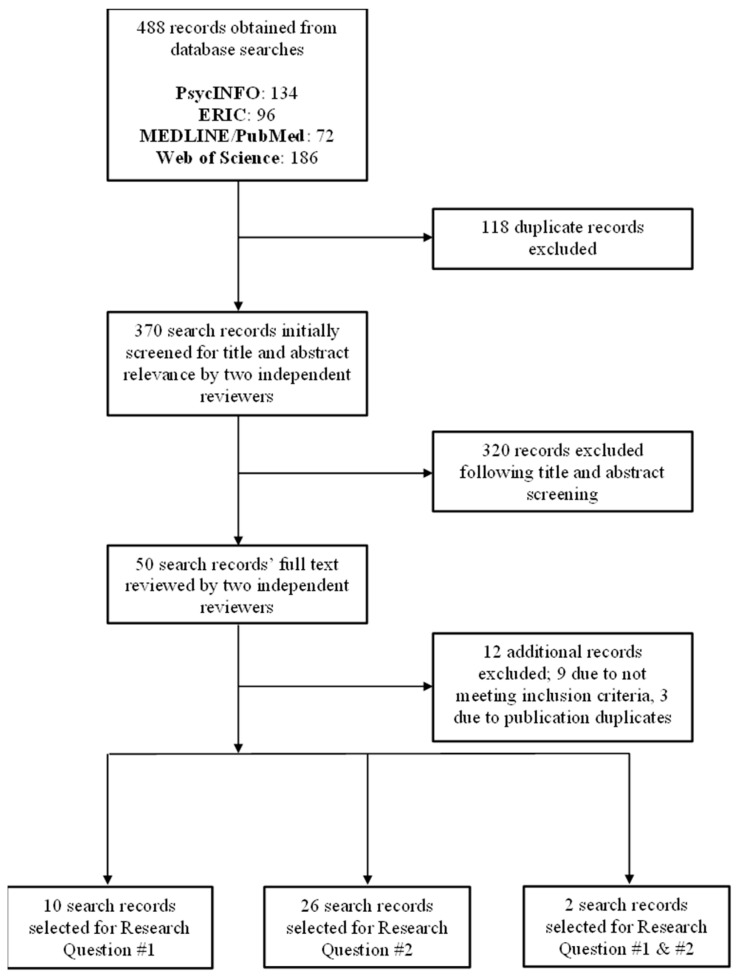
PRISMA-ScR study flow diagram.

Our search yielded 488 initial results. After removing duplicates, 370 articles remained for an initial review of titles and abstracts. Fifty of those articles were eligible for full-text review, nine of which were then excluded because they did not meet the inclusion criteria—specifically, three were excluded because they did not provide specific results for Latino school-aged children, one was excluded because the sample was younger than 6 years, one was excluded because it did not provide data for either cognitive or academic outcomes, and four were excluded because they did not assess physical activity or physical fitness through validated measures. Six articles published similar findings across three separate research studies, so one (duplicate) was removed for each of these respective studies [32,33,34]. Our final sample included 38 articles. Twelve articles were relevant for Research Question #1, 28 articles were relevant for Research Question #2—two articles addressed both research questions and were subsequently included in both questions [35,36]. The screening process for this review can be visualized below in a PRISMA-ScR study flow diagram (Figure 1).

### 3.2. Article and Participant Characteristics

Table 3 includes an overview of the 38 included articles, including their key study and participant characteristics. Most of the included articles reported on studies that were conducted in two countries: the United States (*n* = 18; 47.4%) and Chile (*n* = 17; 44.7%). The remaining studies came from Brazil (*n* = 2; 5.3%) and Colombia (*n* = 1; 2.6%). All studies included Hispanic/Latino youth as a part of their samples, though nearly half (*n* = 16; 42.1%) had mixed samples [24,25,34,37,38,39,40,41,42,43,44,45,46,47,48,49]. The number of participants varied by study from 80 to 187,860 participants, ranging in age from 5.37 years to 17 years. One observational cohort study did include adolescents aged 18 years but examined the prospective association of physical activity and cognitive outcomes at 11, 15, and 18 years of age [50].

#### RQ1: Physical Activity, Physical Fitness, and Cognitive Outcomes

Articles that were related to Research Question #1, “What is the relationship between physical activity or physical fitness with cognitive outcomes in Latino children, aged 6–17?”, were predominately focused on physical activity and/or physical fitness in association with (1) self-concept and mental health outcomes, (2) global cognitive performance, and (3) intellectual ability testing. Twelve articles were identified for this research question—nine had positive study findings, while one reported no relationship. Of the eleven that reported positive associations, two studies [51,52] also reported negative associations between some physical fitness and cognitive outcomes. These studies ranged in publication year from 2015 to 2022, and the majority (*n* = 8; 66.7%) were cross-sectional in design, while the remaining articles were prospective cohort studies (*n* = 2; 16.7%), or randomized controlled trials (*n* = 2; 16.7%). Overall, the articles assessed physical activity with either self-reported questionnaires (*n* = 6; 50.0%) [33,50,52,53,54,55] or accelerometry (*n* = 5; 41.7%) [25,36,45,50,56]. Of these articles, the majority (*n* = 8; 66.7%) also assessed physical fitness—often in combination with physical activity—via the testing of cardiorespiratory fitness [33,36,51,53,55,57], muscular fitness [33,36,51,52,53,55,57], or speed–agility fitness [35,36,51,53,55,57]. 

### 3.3. Physical Activity and Positive Cognitive Outcomes

Several studies reported statistically significant positive associations between self-reported physical activity [33,50,52,53,54,55] or device-based physical activity [25,45,50,56] and various cognitive outcomes. Specifically, these studies found that for school-aged Latino children, higher amounts of physical activity were associated with greater intellectual ability [50,54], global cognitive performance (e.g., working memory, visuospatial memory, psychomotor speed, fluid and logical reasoning, response inhibition, numerical calculation, and selective and divided attention) [33,53,55] and fundamental motor skills (e.g., locomotion, object control, manipulative skills) [45,56]. Two studies also observed that higher amounts of physical activity were associated with higher global self-concept(s) [52] and mental health outcomes [56], important cognitive attributes.

### 3.4. Physical Activity and Cognitive Outcomes—No Associations

One study reported that locomotor skills did not significantly predict school-based moderate-to-vigorous physical activity but did significantly predict sedentary behaviors at school for Hispanic students (β = −0.18, *p* < 0.01) [45]. 

### 3.5. Physical Fitness and Positive Cognitive Outcomes

Physical fitness was primarily assessed via field testing in domains of cardiorespiratory fitness (e.g., 20 m shuttle run) [33,51,53,55,57], muscular fitness (e.g., maximal handgrip strength, long jump test) [33,51,52,53,55,57], and speed–agility fitness (e.g., 4 × 10 m shuttle) [51,53,55,57]. 

Higher cardiorespiratory fitness levels were associated with better global cognitive performance (e.g., working memory, visuospatial memory, psychomotor speed, fluid and logical reasoning, response inhibition, numerical calculation, attention capacity, and selective and divided attention) [33,51,53,55,57] and long-term memory [33]. Similarly, higher muscular fitness levels [33,51,53,55,57] and speed–agility fitness [53,55,57] were also associated with better global cognitive performance. 

Additionally, two randomized controlled trials showed evidence of improvements in cognitive outcomes with improved physical fitness. One of these found that a before-school physical activity intervention for 170 Chilean youths was able to make significantly meaningful changes to both attention and concentration, when cardiorespiratory fitness changed by at least 0.70 mL/kg/min for concentration and at least 3.05 mL/kg/min for selective attention [36]. Another randomized controlled trial found similar significant and positive changes following a 4-month Capoeira intervention for 37 Brazilian children (*n* = 67); executive function (ΔES = 0.59), hand–eye coordination (ΔES = 0.97), and agility (ΔES = 0.39) were most improved in those who had high adherence and greater intervention exposure in the intervention group compared to the control group [35]. 

### 3.6. Physical Fitness and Cognitive Outcomes—No Associations

No significant differences in creativity, selective attention, and concentration were found between those with high and low cardiorespiratory fitness levels—though children with higher cardiorespiratory fitness obtained better scores than their lower fitness counterparts [33]. 

### 3.7. Physical Fitness and Negative Cognitive Outcomes

Two studies found negative associations with specific fitness levels and cognitive outcomes. Specifically, one study found that speed–agility fitness was negatively associated with attention capacity (*p* < 0.05) [51]. Similarly, muscular fitness (i.e., general strength index) was negatively associated with academic (r = −0.09; *p* = 0.02) and total self-concept (r = −0.07; *p* = 0.01). Additionally, it was negatively—though not significantly—associated with social (r = −0.03; *p* = 0.13), emotional (r = −0.06; *p* = 0.17), and family (r = −0.06; *p* = 0.11) self-concept dimensions [52]. 

#### RQ2: Physical Activity, Physical Fitness, and Academic Outcomes

Articles that were related to Research Question #2, “What is the relationship between physical activity, physical fitness, and academic outcomes in Latino children, aged 6–17?”, were predominately focused on physical activity and/or physical fitness in association with academic performance and/or achievement via (1) standardized testing [32,34,35,37,40,41,42,44,47,58,59,60,61], (2) average grade outcomes (e.g., academic grades, grade point average) [25,36,38,46,48,62,63,64,65,66], or (3) academic participation and/or problems [39,43,49]. Two additional articles assessed both standardized testing outcomes and average grades attained during an academic calendar year [24,67]. 

Of these 28 articles, 22 reported significant positive associations. Some of these same studies also reported no relationship (*n* = 5; 17.9%), and three (10.7%) studies reported negative associations—depending on the physical activity and/or physical fitness outcome(s). These studies ranged in publication year from 1992 to 2022, and the majority (*n* = 22; 78.6%) were cross-sectional in design, while the remaining articles were either quasi-experimental (*n* = 1; 3.6%), longitudinal (*n* = 2; 7.1%), repeated-measures crossover (*n* = 1; 3.6%), or randomized controlled trials (*n* = 2; 7.1%).

### 3.8. Physical Activity and Positive Academic Outcomes

Four cross-sectional studies investigated the associations between physical activity and academic performance via standardized testing, reporting that higher physical activity was correlated with better standardized testing results [32,40,41,59]. Specifically, school-aged children with moderate-to-high engagement in physical activity—accumulating 4 h or more of physical activity per week—had significantly higher odds of high academic performance [32,59]. The most consistent association seen across all cross-sectional studies was that higher amounts of physical activity were associated with higher standardized testing results in mathematics and English/language arts [32,40,41,59]. Additionally, one of these studies found that physical activity was strongly and independently associated with developmental reading assessment scores—which was moderately associated with mathematics and language testing scores [40]. 

Three additional cross-sectional studies found favorable associations between physical activity and academic grades. Specifically, school-aged Latino children who either engaged in active transport to school, who attained higher physical activity intensity levels, or who had higher leadership positions within sports had better average grades and performance scores in language, mathematics, reading, and locus of control [39,60,64]. This was especially the case when the physical activity lasted at least 30 to 60 min per session.

A longitudinal study [65] found that, among Hispanic females attending a rural school, participation in any sport was positively associated with senior-level test scores (β = 0.087; *p* < 0.05). Similarly, another study [38] found that interscholastic sports participation was significantly positively associated with end-of-year grade point average outcomes, but not school engagement, for Hispanic students (*p* < 0.05).

### 3.9. Physical Activity and Academic Outcomes—No Associations

Three cross-sectional studies demonstrated inconclusive or null relationships between physical activity and academic outcomes (e.g., standardized testing outcomes, average course grades, academic problems) [24,43,49]. These studies found that both device-based and self-reported physical activity was not significantly associated with academic outcomes; in addition, one of these studies found that being active during weekends was unrelated to academic achievement [43].

### 3.10. Physical Activity and Negative Academic Outcomes

Three cross-sectional studies found evidence of negative associations between physical activity and academic outcomes. One study [46] found that school-aged children (68% Latino) had lower grade point averages when they engaged in higher levels of moderate-to-vigorous physical activity. This finding corroborates those from another study [25], which also found evidence that moderate-to-vigorous physical activity was negatively—though weakly—associated with a core grade point average for English, math, science, and social studies, while very vigorous physical activity was negatively associated with core grade point average—or the unweighted average of all core classes during grades 9 through 12. Additionally, one study [62] observed negative correlations between physical activity practice and average grades—and that physical activity practice was responsible for less than 1% of the variations of grades in students.

### 3.11. Physical Fitness and Positive Academic Outcomes

Six cross-sectional studies assessed physical fitness in association with standardized testing outcomes [37,42,58,60,61,67]. These studies found that school-aged Latino children who attained higher levels of cardiorespiratory fitness had higher odds of having high test scores, predominantly for math and language [37,58,60,61,67]. Additionally, two of these studies found that greater muscular fitness was significantly associated with higher test scores—particularly in math and language, but also in the natural and social sciences [60,61]; and speed–agility fitness elicited a complementary—though not significant—mediating effect on science test scores [67]. 

A prospective cohort study [47] found a significant achievement gap in math standardized test outcomes between both younger Latino students (β = 0.287; *p* < 0.001) and older Latino student (β = 0.168; *p* < 0.001) who went on to pass a series of physical fitness tests and those who failed the physical fitness tests. This evidence corroborates with a quasi-experimental study that found that an in-school, 10–15-min physical activity intervention, paired with cognitively engaged exercises, had statistically significant effects on standardized testing outcomes—with an average 20-point increase in math scores observed for Latino students when compared to Latino students in the control group [34]. 

Three cross-sectional studies investigated the associations between physical fitness and average academic grades [48,66,67]. These studies found that greater cardiorespiratory fitness was significantly associated with better academic grades in reading, writing, mathematics, science, and language scores and performance [48,66,67]. One of these studies [48] also found a significant association between the number of curl-ups performed and the overall average grades of Latino students. 

Evidence from two randomized controlled trials and a repeated-measures crossover study found significant associations between improved physical fitness and academic performance. These studies both found meaningful and statistically significant improvements in academic outcomes—especially language and mathematics—following physical activity interventions ranging from 8 weeks [36] to 4 months [35]. Specifically, significant changes were seen in language (0.63; 95% CI 0.49 to 0.77; ES = 0.83) and mathematics (0.49; 95% CI 0.32 to 0.66; ES = 0.60) performance (*p* < 0.001) in the intervention group [36]. One study [44] conducted a video game-based physical activity intervention for 208 school-aged Latino children. This study found significant improvements in mathematics performance and cardiorespiratory fitness—assessed via 1-mile run times—following an intervention over two academic calendar years, with the most change in cardiorespiratory fitness seen during the first year of the intervention.

### 3.12. Physical Fitness and Academic Outcomes—No Associations

Two studies found an inconclusive or no association between physical fitness and academic outcomes. One study [42] found no evidence to suggest that cardiorespiratory fitness was associated with testing outcomes—specifically in math and English. Additionally, another study [67] found no significant relationship between either muscular fitness or speed–agility fitness with academic (i.e., language) grades, though cardiorespiratory fitness was observed to have a positive association with academic achievement.

## 4. Discussion

### 4.1. Summary of Evidence

This scoping review investigated the associations of both physical activity and physical fitness on cognitive and academic outcomes in school-aged Latino children. Though prior reviews have investigated similar relationships in the general school-aged population, to the authors’ knowledge, this is the first review paper detailing the state of the literature specifically on the school-aged Latino population. This review found evidence to indicate that physical activity and physical fitness are both positively associated with cognitive and academic outcomes for school-aged Latino children. The main findings highlight that higher amounts of self-reported and device-based physical activity, as well as higher physical fitness levels, are associated with greater cognitive performance, motor skills, and long-term memory in school-aged Latino children. Additionally, considerable evidence was found for the associations between physical activity or physical fitness and academic outcomes. 

Most (11/12; 91.7%) of the studies addressing Research Question #1 reported positive findings for (1) physical activity and intellectual ability, global cognitive performance, fundamental motor skills, global self-concept(s), and/or mental health outcomes [33,45,50,52,53,54,55,56] and (2) physical fitness (e.g., cardiorespiratory fitness, muscular fitness, speed–agility fitness) and global cognitive performance and long-term memory [33,35,36,51,52,53,55,57]. Other reviews on this topic have elucidated similar findings pertaining to the positive relationships between physical activity and physical fitness and cognitive performance in school-aged children, regardless of race/ethnicity [68,69]. These findings have even been observed in children as young as 3 years of age [70], thereby showcasing the importance of facilitating physical activity and optimizing physical fitness earlier in the lifespan. Subsequently, the positive findings for Research Question #1 outlay an understanding that physical activity and subsequent physical fitness may be important areas to focus on throughout a Latino child’s life for its potential benefit(s) on cognitive outcomes—especially when considering that cognitive function may impact academic outcomes.

Similarly, most (22/28; 78.6%) of the studies addressing Research Question #2 reported positive findings for (1) physical activity and standardized testing results, academic grades, and grade point average outcomes [32,36,38,39,40,41,59,60,64,65], and (2) physical fitness—namely cardiorespiratory fitness and muscular fitness—and standardized testing results and academic grades [34,35,36,37,42,44,47,48,58,60,61,66,67]. These positive associations were predominantly found with respect to academic outcomes in math and English/language, but associations were also observed in reading and science outcomes. This is consistent with other reviews that concluded that physical activity and physical fitness are both associated with academic outcomes in math, reading, and language in school-aged children, regardless of race/ethnic group [71,72]. Therefore, this review evidences clear and marked positive associations between physical activity and physical fitness on school-aged Latino children’s academic outcomes. 

While markedly fewer in number, several studies (5/38; 13.2%) reported negative associations, and 6/38 (15.8%) reported no relationship between physical activity or physical fitness and cognitive or academic outcomes. These findings corroborate those from a review paper that found mixed associations between physical activity and academic outcomes in school-aged children [73]. Occasionally, the same studies that reported positive associations also reported negative associations, depending on the domain of physical fitness [36,52]. Such findings are likely possible due to the proposed effects of physical activity type on cognitive outcomes. Different forms of acute activity bouts (e.g., aerobic exercise, resistance training) have been observed in randomized controlled studies to elicit differing amounts of changes in adult [74] and adolescent children’s [75] cognitive function(s), with aerobic activities tending to yield more optimal outcomes. Therefore, the type of activity that is performed and the subsequent fitness attained due to the type of activity (e.g., cardiorespiratory, muscular, speed–agility) should be considered as potential explanations for these mixed findings. Furthermore, these findings could also be explained by external factors (e.g., diet, nutrition, sleep, cultural influences) that affect development and cognition from child to child, the effects of which may transcend into academic outcomes as well [76]. 

### 4.2. Gaps and Opportunities for Future Research

A key gap identified through the current review was related to study design. The majority (30/38; 78.9%) of the studies were cross-sectional, while only 4/38 (10.5%) were longitudinal studies, which are vital to understanding these relationships over time—outlining the need for additional studies adopting longitudinal methodologies. For both Research Questions #1 and #2, there were only two studies that applied an experimental design [34,44], and only two studies were randomized controlled trials [35,36]. The interventional studies included in this review paper documented positive associations for both cognitive and academic outcomes. These interventions ranged in duration from 8 weeks up to 2 full academic calendar years, with varying degrees of volume. Because there were few interventions and they varied widely in physical activity dosage, we are unable to draw any conclusions on the interventional and experimental effects of physical activity on cognitive and academic outcomes for school-aged Latino children. The lack of interventional efforts warrants further evaluation, considering the compelling evidence that can be gained regarding specific, tailored approaches for Latino children. Effective interventions are needed to address disparities in overall achievement for school-aged Latino children and optimize health for a population that is at risk of the early development of chronic health conditions [8], which can have serious implications for cognitive outcomes and subsequent academic outcomes, too [7,9].

Another vital gap identified in this review is the total amount of evidence directly addressing RQ1 (*n* = 12) and RQ2 (*n* = 28). The discrepancy in evidence for RQ1 indicates a potential focus for future efforts. Those efforts should urgently address the associations between physical activity and physical fitness with respect to cognitive outcomes—though continued investigation in association with academic outcomes is also encouraged. 

An additional important gap identified in this review is the lack of intensity-specific physical activity data. Very few studies (5/38; 13.2%) assessed physical activity through device-based measures (e.g., accelerometry), which can provide information about physical activity intensities [25,36,45,50,56]. The overwhelming lack of studies reporting intensity-specific data highlights a crucial gap for physical activity and/or physical fitness and cognitive and academic outcomes for school-aged Latino children, as it is understood that the intensity of physical activity has a significant and positive relationship with various health and developmental indicators in school-aged children and youth [77]. 

Future research efforts in this field could be largely beneficial in improving Latino child health and development, while addressing some of the disparities seen in Latino school-aged children’s health. With the gaps identified in this review, key areas of future research should focus on interventional efforts, considering the lack of those focused on the school-aged Latino population. They should be culturally tailored to improve overall engagement in physical activity, thereby optimizing the chances of improving physical health, cognitive, and academic outcomes for this population. The practical implications of this work may help to inform not only the overall comprehension of this literature base, but also guide public health policy promoting physical activity for school-aged Latino children, given the potential benefits.

### 4.3. Strengths and Limitations

This review’s thorough and inclusive search strategy is a major strength. Though most of the studies identified and included in this scoping review were cross-sectional, the inclusion of various study types in the search strategy helps give readers an expansive look into the current state of this literature over a 30+ year timeframe. Additionally, the included studies encompassed a wide range of ages (5.37 to 17 years), delineating the relationship of physical activity and physical fitness with cognitive and academic outcomes throughout primary and secondary school. Such a broad lens is imperative when considering how engagement in physical activity and subsequent physical fitness can augment both cognitive and academic outcomes as children grow and develop. Additionally, though a scoping review, this review implemented a more rigorous and systematic approach in article selection than traditionally done in scoping review methodologies.

This scoping review is not without limitations. Firstly, the review process was facilitated with the use of artificial intelligence in PICO Portal. Though this can accelerate the overall screening process, artificial intelligence can lack the ability to understand the context of the work that is being performed—and the model that is utilized in PICO Portal can contribute to a selection bias, given that it learns keywords from the selected articles that reviewers accept or decline throughout the review process. Secondly, 16 of the 38 articles (42.1%) had mixed race/ethnicity participant samples. Though mixed samples aid our understanding of how school-aged Latino children compare to other racial/ethnic groups, it also limited our ability to tease out the associations for Latino children specifically in those studies. Lastly, the articles came from a handful of countries (i.e., United States, Chile, Brazil, and Colombia). Therefore, the findings may not be generalizable to all Latino school-aged children globally.

## 5. Conclusions

This scoping review found substantial evidence indicating that physical activity and physical fitness are both positively associated with cognitive and academic outcomes in school-aged Latino children. Specifically, engaging in and reporting higher amounts of physical activity and optimizing physical fitness—especially cardiorespiratory fitness—appeared to have significant associations with global cognitive performance and various markers of academic performance and achievement. A significant research gap was elucidated, demonstrating the need for additional physical activity interventional studies for school-aged Latino children. The lack of interventional efforts highlights important next steps for future studies, which should adopt interventional strategies to optimize cognitive and academic outcomes in school-aged Latino populations. The benefits of these interventional efforts may help to improve educational and health equity across the United States and, with sufficient replication, the globe as well.

## Figures and Tables

**Table 1:** Scoping review eligibility criteria.

	Inclusion	Exclusion
Population	Latino/Hispanic school-aged children, ranging from 6 to 17 years of age; studies with children younger than 6 and/or older than 17 may be included if there are also data for participants between 6 to 17 years; Studies in which the majority of the sample (51%+) is Latino/Hispanic; Studies in which the sample is less than 51% Latino/Hispanic children may be included if (1) number and/or percentage of said population are reported; (2) results specific to said population are reported.	Studies with children younger than 6 and/or older than 17 years if data during 6 to 17 years are not provided; Studies that do not have Latino/Hispanic children; Studies in which less than 51% of the sample is from Latino/Hispanic population and do not report number and results.
Intervention	Any form of physical activity–structured and/or free-play (e.g., afterschool programs, sports participation, public health daily recommendations for pediatric physical activity); Studies without interventions may be included if they assess a physical activity-based variable as an independent variable.	Studies that do not have a physical activity-based outcome in place of an intervention.
Comparison	Any physical activity intervention (i.e., afterschool programs, sports, structured physical activity), physical activity-based measure, or physical fitness measure; No intervention or any physical activity or fitness measurement without interventional programming (i.e., accelerometry, physical activity questionnaires, cardiorespiratory fitness testing); Control group.	
Outcomes	Cognition measured through (1) executive function, (2) episodic memory, AND/OR (3) language/vocabulary skills; Academic performance measured through (1) standardized examination AND/OR (2) grade reporting and outcomes.	No measurement of cognition through (1) executive function, (2) episodic memory, AND/OR (3) language/vocabulary skills; No measurement of academic performance through (1) standardized examination AND/OR (2) grade reporting and outcomes.
Study Type	The following study types are applicable: (1) randomized controlled trials, (2) cross-sectional studies (3) cohort studies, (4) case–control studies, (5) qualitative studies; Studies with an English and/or Spanish copy.	The following study types are to be excluded: (1) review papers, (2) protocol papers (3) methods papers, (4) dissertations, (5) report papers, (6) guide papers, (7) textbooks; Studies without an English and/or Spanish copy.

**Table 2:** Sample PubMed search strategy.

Description	Search Terms
Population	(Argentinian or Argentinians or Bolivian or Bolivians or Brazilian or Brazilians or (Central adj1 American *) or chicano or chicanos or Chileans or Colombian or Colombians or (Costa adj1 Rican *) or Cuban or Cubans or Dominican or Dominicans or Ecuadorians or Guatemalan or Guatemalans or hispanic or hispanics or Honduran or Hondurans or latin or latina or latinas or latino or latinos or latinx or mestizo or mestizos or mexican or mexicans or Nicaraguan or Nicaraguans or Panamanian or Panamanians or Paraguayan or Peruvians or (Puerto adj1 Rican *) or Salvadoran * or Uruguayan or Venezuelan or Venezuelans or (spanish adj2 speak *))
Age	AND (Child OR children OR adolescent OR adolescence OR “school age” OR teen OR teenager OR tween OR youth)
Physical activity	AND (“physical activit *” OR “physical fitness” OR “weight training” OR “resistance training” OR “aerobic” OR exercise * OR “weight lifting”)
Cognitive and academic outcome(s)	AND (“executive function *” OR “working memory” OR “cognitive flexibility” OR “inhibitory control” OR “episodic memory” OR (learning AND recall) OR “language skills” OR vocabulary OR academic)

*: a Boolean operator. Boolean operators provide search results that contain variations of the root word. This helps to maximize the number of potential articles that are included during the search process.

**Table 3:** Study characteristics.

Author and Year	Study Design	Sample Size, Age, and Country	Intervention	Duration	Physical Activity/Fitness Measure(s)	Cognitive Outcome Measure(s)	Academic Outcome Measure(s)	Results
Behringer et al., 2022 [29]	Cross-sectional	*n* = 4936 USA	Not applicable	Not applicable	MVPA assessed with accelerometers (ActiGraph wGT3X-BT); at least 3 wear days necessary for valid data	Not applicable	Academic achievement assessed with course grades for math, reading, spelling, and standardized test scores in writing, math, reading, and Lexile	MVPA and academic achievement was found to be moderated by student Hispanic ethnicity for one of the 15 academic achievement outcomes measured, Grade 4 fall spelling marks (β = −0.159; *p* < 0.0001)
Cosgrove and Castelli, 2018 [30]	Cross-sectional	*n* = 806 14.56 ± 1.15 years USA	Not applicable	Not applicable	PF evaluated with the FITNESSGRAM; PA evaluated with accelerometers (Actigraph GT3X)	Not applicable	Academic performance assessed with a grade point average from student final grade averages for English, math, science, and social studies classes	Only time spent in very vigorous PA had a statistically significant (*p* < 0.05) association (negative) with academic performance
Burrows et al., 2014 [37]	Cross-sectional	*n* = 1271 Low-activity group: 12.9 ± 2.3 years Moderate-activity group: 14.7 ± 1.9 years High-activity group: 15.3 ± 1.5 years Chile	Not applicable	Not applicable	Weekly hours for scheduled PA assessed with a validated questionnaire	Not applicable	Academic achievement assessed with the SIMCE; focused specifically on language, mathematics, and science	Devoting more than 4 h/week to scheduled PA significantly increased (*p* < 0.01) the odds of having SIMCE composite z-scores ≥ 50th percentile and ≥75th percentile
Caamaño-Navarrete et al., 2021 [38]	Cross-sectional	*n* = 248 Boys: 11.80 ± 1.17 years Girls: 11.58 ± 1.09 years Chile	Not applicable	Not applicable	PF evaluated with Léger Test; PA assessed using the PAQ-C	Creativity assessed with CREA test; memory was assessed with Ray’s Auditory Verbal Learning Test; attention capacity was assessed with the d2 Test of Attention	Not applicable	Creativity was positively associated (*p* < 0.05) with max oxygen consumption; long-term memory was positively associated (*p* < 0.05) with CRF
Hollar et al., 2010 [39]	Quasi-experimental	*n* = 1197 7.85 ± 1.67 years USA	Dietary intervention; holistic nutrition and healthy lifestyle curricula; increased PA during the school day/classroom	2 school years	PA intervention consisted of increased activity in the classroom in bouts of 10 to 15min desk-side activity (WISERCISE!) matched with core academic areas	Not applicable	Academic achievement assessed with the FCAT administered in the third grade	Intervention children had significantly higher FCAT math scores than the control (*p* < 0.001); Hispanic children in the intervention had >20-point gain in math scores than Hispanic children in the control (*p* = 0.006)
Fernandes et al., 2022 [40]	Randomized Controlled Trial	*n* = 67 (37 experimental group; 30 control) Brazil	Capoeira PA intervention (dynamic, involving simple multi-limb movements); 90 min sessions	4 months	A standard agility test was utilized to assess speed–agility	EF subsets of the Wechsler Intelligence Scale for Children were used to assess cognitive function; motor skills also assessed with hand–eye coordination tests	Academic achievement assessed with a standardized academic achievement test	A positive dose–response relationship between number of classes attended and improvement in EF composite (*p* < 0.01) was observed; large effect seen in the HG EG for academic achievement (ΔES = 0.37)
García-Hermoso et al., 2020 [41]	Randomized Controlled Trial	*n* = 170 10.18 ± 0.84 years Chile	Before-school PA intervention of various active games; 5× per week	8 weeks	PF evaluated with the ALPHA Health-Related Fitness Test Battery for Children and Adolescents; MVPA assessed with accelerometers (GENEActiv tri-axial accelerometer)	Attention capacity was assessed with the d2 Test of Attention	Academic performance assessed using the children’s grades in core subjects (mathematics and language)	Significant changes were seen in language and mathematics performance (*p* < 0.001); a significant relationship between interventional effects and attention and concentration when change in CRF was above, but not below, 3.05 and 0.70 mL/kg/min
Aske et al., 2018 [42]	Cross-sectional	*n* = 1423 USA	Not applicable	Not applicable	CRF evaluated using an age and sex-adjusted 20 m shuttle run	Not applicable	Academic performance assessed with MCAS math and ELA z-scores	CRF was positively associated with ELA MCAS (*p* < 0.05) and with math MCAS (*p* < 0.01); Black and Latino students performed lower in ELA (*p* < 0.001) and math MCAS (*p* < 0.001) than White students
Bang et al., 2020 [43]	Cross-sectional	*n* = 16,200 USA	Not applicable	Not applicable	PA was assessed through self-reporting of sport participation	Not applicable	Academic achievement assessed with 12th grade academic GPA	Hispanic sport participation was not significant for their school engagement but significant (*p* < 0.05) for GPA; Hispanic non-sport participants’ school engagement was significantly (*p* < 0.01) higher, but they had lower GPAs than White non-sport participants
Bang et al., 2019 [44]	Cross-sectional	*n* = 7217 USA	Not applicable	Not applicable	PA was assessed through self-reporting of sport participation	Not applicable	Academic performance was assessed with reading and math performance via item response theory	Leader sport participants who were Hispanic demonstrated significantly higher scores in locus of control (*p* < 0.05), reading (*p* < 0.01), and math (*p* < 0.01) than sport participants and non-sport participants
Caldas and Reilly, 2019 [45]	Cross-sectional	*n* = 526 USA	Not applicable	Not applicable	PA assessed using the PAQ-C	Not applicable	Academic outcomes assessed with New York ELA and math student scaled scores	PA mediated reading levels and ELA and math scores (*p* < 0.001)—particularly for Hispanic students (*p* < 0.001)
Caldas and Reilly, 2018 [46]	Cross-sectional	*n* = 964 USA	Not applicable	Not applicable	PA assessed with the PAQ-C for weekly average PA levels	Not applicable	Academic achievement assessed with student scaled scores on the spring 2012 New York State ELA and mathematics tests	PA had a significant (*p* < 0.001) and substantive and positive effect on ELA achievement; the effects of PA on mathematics were significantly (*p* < 0.001) stronger than those in ELA for Hispanic children
Cosgrove et al., 2018 [47]	Cross-sectional	*n* = 397 14.23 ± 1.85 years USA	Not applicable	Not applicable	PF evaluated with the FITNESSGRAM—PACER test	Not applicable	Academic performance assessed by averaging year-end score on state academic accountability subject tests	PACER results were not significantly associated with academic performance
Galindo-Perdomo et al., 2021 [48]	Cross-sectional	*n* = 2624 13.7 ± 1.4 years Colombia	Not applicable	Not applicable	PA assessed with a 7-day PA recall	Not applicable	Academic achievement assessed with questionnaire on academic history of failure/passing	Academic achievement variables did not reduce or increase the likelihood of being active during weekdays; boys reported worse academic achievement (*p* < 0.05) while engaging in more sports clubs and non-organized PA (*p* < 0.05)
Gao et al., 2013 [49]	Repeated-measures Crossover	*n* = 208 10.32 ± 0.91 years USA	DDR-based exercise program; 3× per week	2 school years	Cardiorespiratory endurance assessed with the 1-mile run test	Not applicable	Academic performance assessed with the Utah Criterion-Referenced Test for math and reading scores	Significant changes in CRF via 1-mile run were observed between intervention years 1 and 2 for students starting out in 3rd grade (*p* = 0.009); intervention children had greater improvement in math scores than comparison children in Year 1 and Year 2 (*p* < 0.05)
Gu et al., 2019 [50]	Cross-sectional	*n* = 671 6.97 ±1.6 years USA	Not applicable	Not applicable	MVPA and SB were assessed with accelerometers (Actical Monitors) during the school day—5-day period	PE Metrics FMS assessment was used to assess fundamental motor skills	Not applicable	In Hispanic children, object-control but not locomotor skill significantly (*p* < 0.01) predicted school-based MVPA
Huang et al., 2012 [51]	Cross-sectional	*n* = 666 12.5 ± 0.66 years USA	Not applicable	Not applicable	PA assessed with modified version of the previous day PA recall	Not applicable	Academic performance assessed with students’ self-reported grades in the last year on a 4.0 scale; compared to GPA from prior school year	Both measured and self-reported grades decreased with blocks of MVPA time (*p* < 0.05)
London and Castrechini, 2011 [52]	Cross-sectional, Longitudinal	Cohort 1: *n* = 1325 Cohort 2: *n* = 1410 USA	Not applicable	Not applicable	PF evaluated with the California PFT–FITNESSGRAM	Not applicable	Academic outcomes assessed with CST in math and ELA	Cohort 1: Students who passed both PF tests and students who pass in the second year have significantly higher math scores in the 4th grade compared to students failed both (β = 0.244 and β = 0.147, respectively) Cohort 2: The CST gap only occurs for students who passed both PFTs (β = 0.218 for math; β = 0.154 for ELA)
Lorenz et al., 2017 [53]	Cross-sectional	*n* = 80 9.39 ± 0.52 years USA	Not applicable	Not applicable	PF evaluated with the FITNESSGRAM protocol	Not applicable	Academic achievement assessed by compiling standardized teacher-assigned grades in reading, writing, mathematics, social studies, and science during the third-quarter report cards	Aerobic fitness had significant influence on reading (*p* < 0.05), writing (*p* < 0.004), mathematics (*p* < 0.030), and science (*p* < 0.020) grades
Shi et al., 2014 [54]	Cross-sectional	Boys: *n* = 1559 Girls: *n* = 1342 USA	Not applicable	Not applicable	The community-wide Children’s Health Assessment and Planning Survey was administered to assess children’s health status and self-reported PA	Not applicable	Academic performance assessed with questions on academic problems (yes or no)	Children’s academic problems were marginally associated with physical activity level, but not statistically significant
Esteban-Cornejo et al., 2015 [55]	Cohort	*n* = 3235 Brazil	Not applicable	Not applicable	PA assessed with IPAQ and accelerometer (GENEActive) at 18 years of age	Cognitive performance assessed using Brazilian version of the Wechlser Adult Intelligence Scale	Not applicable	Self-reported PA was positively associated (*p* < 0.001) with cognitive performance at 18 years of age; higher levels of activity were associated with higher cognitive performance scores (*p* < 0.05)
García-Hermoso et al., 2020 [56]	Cross-sectional	*n* = 201 Girls: 12.28 ± 2.14 years Boys: 11.66 ± 1.84 years Chile	Not applicable	Not applicable	PF evaluated with ALHPA-fitness and FUPRCOL test batteries	Attention capacity was assessed with the d2 Test of Attention	Not applicable	Attention capacity was positively associated with MF (*p* < 0.001), CRF (*p* < 0.001), S-AF (*p* = 0.027), and overall fitness (*p* = 0.004)
Ferrari et al., 2022 [57]	Cross-sectional	*n* = 1078 9.1 ± 1.1 years Chile	Not applicable	Not applicable	PA assessed using the PAQ-C—Spanish version 2; upper limb muscular strength assessed using hydraulic dynamometer (Jamar^®^ PC-5030); lower limb muscular strength assessed with standing long jump	Self-concept assessed using Five-Factor Self Concept Questionnaire	Not applicable	PA was significantly associated with academic (*p* = 0.030), social (*p* = 0.04), family (*p* = 0.01), physical (*p* = 0.01), and total self-concepts (*p* = 0.01); upper limb strength was negatively associated with academic (*p* = 0.01) and total self-concepts (*p* = 0.01)
Hernández-Jaña et al., 2021 [58]	Cross-sectional	*n* = 1196 11.71 ± 1.06 years Chile	Not applicable	Not applicable	PF evaluated with ALHPA-fitness test battery	The NeuroCognitive Performance Test used to assess cognitive performance	Not applicable	CRF, MF, S-AF, and GFS were all significantly (*p* < 0.05) associated with cognitive performance
Hormazabal-Peralta et al., 2018 [59]	Cross-sectional	*n* = 79 16 ± 1.19 years Chile	Not applicable	Not applicable	Weekly PA assessed using a validated questionnaire	HIA assessed using Raven’s progressive matrices general scale	Not applicable	Most (69.86%) of HIA students engaged in more than 2 h of PA per week; students who engaged in more than 2 h of PA showed lower FMI and BF% (*p* < 0.05) and higher MM% (*p* < 0.05)
Lemes et al., 2021 [60]	Cross-sectional	*n* = 1196 12.21 ± 1.05 years Chile	Not applicable	Not applicable	PF evaluated with ALPHA-fitness test battery; PA assessed using the 7-day recall YAP-SL	The NeuroCognitive Performance Test used to assess cognitive performance	Not applicable	Fitness was positively (*p* = 0.001) associated with boys’ and girls’ cognitive performance
Gu et al., 2018 [61]	Cross-sectional	*n* = 141 5.37 ± 0.48 years USA	Not applicable	Not applicable	MVPA and SB were assessed with accelerometers (Actical Monitors) during the school day—5-day period	Psychosocial health assessed with the self-report pediatric QoL inventory Spanish short form; PE Metrics FMS assessment was used to assess fundamental motor skills	Not applicable	Locomotor, manipulative, and motor control index variables were all significantly (*p* < 0.05) associated with MVPA; MVPA significantly related to mental health outcomes (*p* < 0.01)
Solis-Urra et al., 2020 [62]	Cross-sectional	*n* = 1171 12.23 ± 1.04 years Chile	Not applicable	Not applicable	PF evaluated with ALPHA-fitness test battery	The NeuroCognitive Performance Test used to assess cognitive performance	Not applicable	Significant main effect for CRF (*p* ≤ 0.001) and MF (*p* = 0.002) on domains of CF, WM, IC, and IN
Ahumada-Padilla et al., 2020 [63]	Cross-sectional	*n* = 12,338 13.5 ± 0.5 years Chile	Not applicable	Not applicable	PF evaluated with the SIMCE test for physical education	Not applicable	Academic performance assessed with standardized tests on reading, mathematics, natural sciences, history and geography, and social sciences	Better global PF was significantly (*p* < 0.001) associated with more possibilities of sufficient level in each of the subjects evaluated for both boys and girls; girls with better global PF had 34% more chance of good academic performance and boys had 21% more
Correa-Burrows et al., 2017 [64]	Cross-sectional	*n* = 1271 Low-activity group: 12.9 ± 2.3 years Moderate-activity group: 14.7 ± 1.9 years High-activity group: 15.3 ± 1.5 years Chile	Not applicable	Not applicable	Weekly hours for scheduled PA assessed with a validated questionnaire	Not applicable	Academic achievement assessed with the SIMCE; focused specifically on language and mathematics	Devoting more than 4 h/week to scheduled PA significantly increased (*p* < 0.01) the odds of reaching the official and discretionary sufficiency in both language and mathematics
García-Hermoso et al., 2017 [65]	Cross-sectional	*n* = 36,870 13.8 ± 0.7 years Chile	Not applicable	Not applicable	PF evaluated with Léger Test; lower limb muscular strength assessed with standing long jump	Not applicable	Academic achievement assessed with the SIMCE used to measure language and mathematics	Adolescents with high levels of CRF and MF had significantly higher academic achievement levels (*p* < 0.001)
Olivares and García-Rubio, 2016 [66]	Cross-sectional	*n* = 18,746 13.8 ± 0.7 years Chile	Not applicable	Not applicable	PF evaluated with the SIMCE test for physical education	Not applicable	Academic performance assessed with 4 standardized tests in language, mathematics, natural sciences, and social sciences	All fitness components were statistically associated (*p* < 0.001) with academic performance; cardiorespiratory capacity was not associated with social science outcomes
Cid et al., 2019 [67]	Cross-sectional	*n* = 713 14.1 ± 1.2 years Chile	Not applicable	Not applicable	PA and sports habits were assessed with a modified version of the Honey–Alonso’s questionnaire	Not applicable	Academic performance assessed with average grades obtained by students	Low and negative correlations observed between active style and all average grades were observed; PA practices were responsible for less than 1% of the variations of grades in students
García-Hermoso et al., 2017 [68]	Cross-sectional	*n* = 389 12.0 ± 0.6 years Chile	Not applicable	Not applicable	PA assessed with a Spanish version of the PAQ-A	Not applicable	Academic achievement assessed using students’ grades in mathematics and language	Academic achievement was higher in adolescents with 30 to 60 min of ACS than non-commuters in language (*p* = 0.016) and mathematics (*p* = 0.031)
García-Hermoso and Marina, 2017 [69]	Cross-sectional	*n* = 395 12.2 ± 0.6 years Chile	Not applicable	Not applicable	PA assessed with the PAQ-A	Not applicable	Academic achievement assessed using students’ grades in core subjects (mathematics and language)	Students classified as obese/low–medium PA (boys: *p* = 0.039; girls: *p* = 0.035) and obese/low–medium PA/excessive screen time (boys: *p* = 0.015; girls: *p* = 0.028) were less likely to have high academic achievement compared to students classified as non-obese/high PA/met screen time recommendations
Melnick et al., 1992 [70]	Cross-sectional, Longitudinal	*n* = 30,000 USA	Not applicable	Not applicable	PA assessed with questionnaires that asked questions regarding athletic participation	Not applicable	Academic performance assessed with school transcripts for half of the 1980 sophomore cohort and standardized testing	Statistically significant coefficients for sports participation and academic performance appeared for Hispanic females attending rural schools (−0.95; *p* < 0.05)
Santiago et al., 2013 [71]	Cross-sectional	*n* = 155 10.7 ± 0.7 years USA	Not applicable	Not applicable	PF evaluated with the FITNESSGRAM—PACER test	Not applicable	Academic achievement assessed with reading and math final percentage grades	Math grades were significantly associated with aerobic capacity in girls (*p* = 0.022); aerobic capacity was found to be a significant predictor of math performance in girls (*p* = 0.016)
Gajardo-Araya et al., 2022 [72]	Cross-sectional	*n* = 1296 11.89 ± 1.19 years Chile	Not applicable	Not applicable	PF evaluated with ALPHA-fitness test battery	Not applicable	Academic performance assessed by having students self-report their grades in the last year on a 4.0 scale; actual GPA was obtained from prior academic year to compare	CRF and GFS were both significantly associated with math, language, and science grades (*p* < 0.001); MF was significantly associated only with math and science grades (*p* < 0.001); S-AF was significantly associated with math (*p* < 0.001) and science (*p* < 0.01)

Abbreviations: ACS: active commuting to school; BF%: body fat percentage; CF: cognitive flexibility; CRF: cardiorespiratory fitness; CST: California standardized test; DDR: Dance Dance Revolution; EF: executive function; ELA: English language arts; FCAT: Florida Comprehensive Achievement Test; FMI: fat mass index; FMS: fundamental motor skills; GFS: global fitness score; HIA: high intellectual ability; IC: inhibitory control; IN: intelligence; IPAQ: International Physical Activity Questionnaire; MCAS: Massachusetts Comprehensive Assessment System; MF: muscular fitness; MM%: muscle mass percentage; MVPA: moderate-to-vigorous physical activity; PA: physical activity; PAQ-A: Physical Activity Questionnaire for Adolescents; PAQ-C: Physical Activity Questionnaire for Older Children; PF: physical fitness; PFT: physical fitness test; QoL: quality of life; S-AF: speed–agility fitness; SB: sedentary behavior; SIMCE: Sistema de Medición de la Calidad de la Educación (Education Quality Measurement System, SIMCE); WM: working memory; YAP-SL: Youth Activity Profile Questionnaire.

## Data Availability

No new data were created or analyzed in this study. Data sharing is not applicable to this article.
